# Nausea and vomiting in pregnancy – association with pelvic girdle pain during pregnancy and 4-6 months post-partum

**DOI:** 10.1186/s12884-018-1764-7

**Published:** 2018-05-08

**Authors:** Arthur Chortatos, Per Ole Iversen, Margaretha Haugen, Malin Eberhard-Gran, Elisabeth Krefting Bjelland, Marit B. Veierød

**Affiliations:** 1 Oslo Centre for Biostatistics and Epidemiology, Department of Biostatistics, Institute of Basic Medical Sciences, University of Oslo, PO Box 1122, Blindern, N-0317 Oslo, Norway; 2 Department of Nutrition, Institute of Basic Medical Sciences, University of Oslo, PO Box 1046, Blindern, N-0317 Oslo, Norway; 3 Department of Hematology, Oslo University Hospital, PO Box 4950, Nydalen, N-0424 Oslo, Norway; 4 Domain for Infection Control and Environmental Health, Norwegian Institute of Public Health, PO Box 4404, Nydalen, N-0403 Oslo, Norway; 5 Domain for Mental and Physical Health, Norwegian Institute of Public Health, PO Box 4404, Nydalen, N-0403 Oslo, Norway; 6 Health Services Research Unit, Akershus University Hospital, PO Box 1000, N-1478 Lørenskog, Norway; 7 Institute of Clinical Medicine, Campus Ahus, University of Oslo, Lørenskog, Norway; 8 Department of Obstetrics and Gynecology, Akershus University Hospital, PO Box 1000, N-1478 Lørenskog, Norway

**Keywords:** Nausea and vomiting in pregnancy, Pelvic girdle pain, Pregnancy hormones, MoBa

## Abstract

**Background:**

To better understand previous associations reported regarding nausea and vomiting in pregnancy (NVP) and pelvic girdle pain (PGP), an investigation into timing of symptom onset for NVP and PGP in pregnancy, as well as the association of NVP with PGP 4-6 months post-partum was performed. We hypothesised that women with NVP symptoms would be most susceptible to experiencing persistence of PGP post-partum.

**Methods:**

Fifty two thousand six hundred seventy-eight pregnancies from the Norwegian Mother and Child Cohort Study were analysed regarding nausea, vomiting, pelvic girdle pain, and health outcome data collected from questionnaires answered between gestation weeks 15, 20, 30, and 6 months post-partum. Logistic regression was used.

**Results:**

Women experiencing NVP and PGP together (6.9%) were heaviest in the sample, youngest at menarche and had highest proportion with education ≤12 years. The primiparous women in this group had the lowest timespan from menarche to pregnancy. Women with nausea alone (NP) and NVP had higher odds of PGP 4-6 months post-partum (adjusted odds ratio, aOR = 2.14, 95% CI 1.70–2.71, and aOR = 2.83, 95% CI 2.25–3.57, respectively), compared to symptom-free women. NP/NVP symptoms appeared early in the first trimester, while PGP symptoms appeared later in pregnancy. Women with longer durations of nausea and/or vomiting had a higher proportion of PGP compared to shorter duration women.

**Conclusions:**

Women with NP and NVP had increased odds of PGP 4-6 months post-partum, and women with a long duration of nausea and/or vomiting had a higher proportion of PGP than women with shorter duration, both during pregnancy and 4-6 months post-partum. This finding suggests a synergistic relationship between NP/NVP and PGP.

**Electronic supplementary material:**

The online version of this article (10.1186/s12884-018-1764-7) contains supplementary material, which is available to authorized users.

## Background

Nausea alone (NP) or nausea and vomiting in pregnancy (NVP) affect approximately 80% of pregnancies [1–3]. Symptoms usually emerge early in the first trimester, around gestational weeks 5-6, peaking around week 9, and subsiding by approximately gestational week 12 or later [1, 4]. Women with a young age, previous deliveries, those overweight or obese, with an early menarche, with premenstrual symptoms, and non-smokers have been associated with symptoms of NP or NVP [5–9]. In addition, women who experienced symptoms in previous pregnancies seem more susceptible to NP or NVP in following pregnancies [10].

Pelvic girdle pain (PGP) is a pregnancy-related complication in which women have pain located between the posterior iliac crest and the gluteal fold, particularly close to the sacroiliac joints and the pubic symphysis [11]. The prevalence is estimated at 20% [11], however some researchers have reported higher prevalence values [12, 13]. The onset of symptoms occur from approximately week 6 of the pregnancy, and reach peak pain intensity between the 24th and 36th week of pregnancy [14, 15]. In previous studies, approximately 10% of women with PGP during pregnancy continued to experience pain up to 18 months after delivery [16, 17]. Known risk factors for PGP are smoking during pregnancy, a high BMI, young maternal age, previous pregnancies, and early menarche [18–23].

We recently reported that women with NP and NVP had increased odds for experiencing PGP compared with symptom-free (SF) women [24]. The NP/NVP and PGP conditions share similar risk factors, occur in the first trimester (particularly NP/NVP), and have unknown aetiologies. The current literature suggests that pregnancy hormones are one of the most likely causes of NVP, with human chorionic gonadotropin (hCG) as the main candidate, although estrogen, progesterone and relaxin have also been discussed [25]. Likewise, PGP aetiology has been linked previously to ovarian hormone levels, since these hormones may influence ligament laxity of the pelvic joints and lead to pain [11, 26]. As so little is understood about NP, NVP and PGP, an investigation of women with both of these conditions may reveal common traits that will provide a better understanding of their nature.

As we have already reported upon the association between NP/NVP and PGP during pregnancy, the aim of the present study was to focus upon women experiencing both NP/NVP and PGP during pregnancy, paying particular attention to the timing of onset for either condition, as well as to investigate PGP prevalence 4-6 months post-partum.

## Methods

### The Norwegian Mother and Child Cohort Study (MoBa)

MoBa is a prospective population-based pregnancy cohort study conducted by the Norwegian Institute of Public Health [27]. Participants were recruited from all over Norway from 1999 to 2008 and 40.6% of all women that were invited consented to participate. The cohort now includes 114,500 children, 95,200 mothers, and 75,200 fathers. Three questionnaires were answered during pregnancy, with follow-up questionnaires answered post-partum at regular intervals. Pregnancy and birth records from the Medical Birth Registry of Norway (MBRN) were linked to the MoBa cohort [28]. The quality-assured data files entitled ‘version 8’ were used. Response rates for the MoBa version 8 questionnaires can be found elsewhere [29, 30].

### Questionnaires and variables

The data were primarily collected from four self-administered questionnaires (Q). The first one was answered in gestational week 15 (Q1), and the subsequent questionnaires were administered around week 20 (Q2), week 30 (Q3), and 6 months post-partum (Q4).

Q1 probed into details regarding maternal health and lifestyle, demographics, previous pregnancies, and early first trimester reports of NP, NVP, and PGP.

From Q1 we used maternal height (cm) and weight (kg) at the start of pregnancy to calculate body mass index (BMI, kg/m^2^), education (seven categories collapsed into: ≤12 years, 13–16 years, or ≥ 17 years), smoking during pregnancy (no, sometimes, or daily), age at menarche (years), incidences of irritability before menses (five categories collapsed into ‘yes/no’), number of previous pregnancies, previous experiences of NVP (yes or no), and experiences of NP, NVP, and PGP in the first 15 weeks of pregnancy (separated into 4 week intervals; yes or no). The incidences of irritability before menses were defined as having reported an alleviation of such symptoms after menses. Years from menarche to pregnancy were calculated by subtracting age at menarche (from Q1) from maternal age at time of birth (from MBRN). Due to obvious erroneous reporting, only values ≥5 years and < 35 years from menarche to current pregnancy were used. Likewise, ages at menarche < 7 years and > 25 years of age were considered to be erroneously reported.

Q2 included a food frequency questionnaire and was answered in mid-pregnancy. Version two of Q2 included detailed questions regarding nausea and vomiting, therefore only women answering this version were included in the sample. While the questions regarding NVP and PGP in Q1 were structured simply as ‘yes/no’ for the corresponding 4 week intervals in early first trimester, the NVP questions in Q2 probed experiences of nausea or vomiting during pregnancy (yes or no), the gestational week of onset and cessation, and whether women were still experiencing nausea and/or vomiting at the time of answering the questionnaire (around gestational week 20). These NP and NVP answers were cross-checked with answers provided in Q1 to avoid inconsistent responses, as detailed in the ‘Study Sample’ section below. Prior to calculating the duration of either nausea or vomiting from variables stating the gestational week of onset and the week of cessation of these conditions, recoding for some variables was required, as described in detail previously [5].

Data from Q3 (gestational week 30) enabled us to differentiate NVP women from women diagnosed with hyperemesis gravidarum (HG), defined as prolonged nausea and vomiting during pregnancy requiring hospitalisation before week 25 of gestation [31]. Q3 also provided comprehensive information regarding pain distribution and pain intensity (none/mild/severe) in the pelvic girdle during pregnancy. We defined PGP as self-reported pain in both the pubic symphysis in the anterior pelvis and bilaterally in the sacroiliac joints in the posterior pelvis (yes or no). Severe PGP (sPGP) was defined as having reported severe pain in all the above pain locations [21]. These conditions are referred to as PGP and sPGP, respectively. We also obtained responses regarding experiences of NP, NVP, and PGP from week 15 to week 29 or more of pregnancy (separated into 4 week intervals; yes or no). All ‘yes/no’ responses for NP, NVP, and PGP from Q1 and Q3 were cross-checked with identified cases based upon the more comprehensive definitions of NP, NVP, and PGP defined above.

We based the reporting of PGP or sPGP during the last part of pregnancy and/or 4-6 months post-partum upon questions in Q4 (six months post-partum) regarding pain in the anterior and bilateral posterior pelvis [32]. Women reporting PGP or sPGP during the last part of pregnancy were added to those women reporting PGP or sPGP in Q3, producing the total number of women reporting PGP or sPGP during pregnancy.

Data concerning maternal age at time of birth (year), parity (primiparous, multiparous), and gestational length (weeks) were obtained from the MBRN. Questions allowing only for an answer of ‘yes’ had non-answers recoded to ‘no’ or ‘none’ from MoBa and MBRN [24].

### Study sample

The entire MoBa cohort includes 114,275 children and 95,200 mothers. The present sample comprises those women deemed eligible for inclusion, having satisfied criteria such as answering relevant questions related to the study in Q1 and version 2 of Q2, being registered in the MBRN with a singleton live birth, with complete weight and height data recorded, with a gestational length between 28 to 42 weeks, and with consistent reports regarding symptoms of NP and NVP throughout the first trimester. Furthermore, as some women participated in MoBa multiple times due to multiple pregnancies, only those women with one participation were included, or else only the first pregnancy if more than one was registered. Women with NP or NVP symptoms were only included if their condition did not develop into HG. A total of 52,678 women fulfilled all these criteria and were included in the study sample. These criteria were identical to those used in our previous studies [5, 24, 33].

### Statistical analyses

The study sample was divided into three groups: symptom-free (SF), nausea alone (NP), and both nausea and vomiting (NVP). Furthermore, we categorised the women according to whether they were with or without all forms of PGP during pregnancy. Two variables were dichotomised according to the median value: nausea duration (< 8 or ≥ 8 weeks) and vomiting duration (< 7 or ≥ 7 weeks). Only women reporting either NP, NVP, or PGP both in the simple ‘yes/no’ questions as well as the more detailed definitions used for these conditions were included in the analyses regarding the reported week of onset.

Descriptive results are presented as means (standard deviations; SDs) or frequencies (%). Comparisons between women with or without PGP, stratified by condition (SF, NP and NVP), were performed by two sample t-test and chi-squared test. PGP and sPGP at 4-6 months post-partum were explored using logistic regression analysis. We present crude and adjusted odds ratios (cOR and aOR, respectively), with 95% confidence intervals (CI). We adjusted for maternal age, BMI, smoking during pregnancy, parity, education, age at menarche, and incidences of irritability before menses after reviewing previous literature regarding NP, NVP, and PGP. Additional adjustment for length of gestation, years from menarche to pregnancy, and use of hormonal contraceptives prior to pregnancy did not change the results, therefore these results are not reported.

A significance level of 0.05 was used except when we studied statistical interaction effects, where 0.01 was used to allow for multiple testing. All analyses were performed using SPSS 22.0 (IBM Corp, Armonk, NY, USA). We followed the guidelines in the Strengthening the Reporting of Observational Studies in Epidemiology (STROBE) statement (http://www.strobe-statement.org).

## Results

### Maternal and gestational history

Women with PGP tended to be heavier and with a higher pre-pregnancy BMI compared to those without PGP (Table 1), and the highest means were for NVP women with PGP. Age at menarche and the number of years from menarche to pregnancy were slightly lower for SF, NP, and NVP women with PGP as compared to women without PGP. SF, NP, and NVP women with PGP had the highest proportion with an education ≤12 years, the highest proportion of irritability before menses amongst those primiparous, and the highest proportion with previous experiences of NVP amongst those multiparous, compared to women without PGP.

**Table 1:** Maternal characteristics for symptom-free (SF) women, women with nausea alone (NP), and women with nausea and vomiting (NVP) according to the presence of pelvic girdle pain^a^ (PGP) during pregnancy, *n* = 52,678

		SF			NP			NVP		
		no PGP (*n* = 13,045)	PGP (*n* = 1456)	*P* value	no PGP (*n* = 16,908)	PGP (*n* = 3690)	*P* value	no PGP (*n* = 13,953)	PGP (*n* = 3626)	*P* value
	*n*	Mean (SD)	Mean (SD)		Mean (SD)	Mean (SD)		Mean (SD)	Mean (SD)	
Maternal age at delivery (years)	52,678	30.3 (4.6)	30.0 (4.7)	0.06	30.8 (4.5)	30.7 (4.4)	0.06	29.1 (4.7)	29.2 (4.7)	0.60
Maternal weight at delivery (kg)	52,678	67.2 (12.1)	69.9 (13.6)	< 0.001	67.3 (11.9)	69.8 (13.4)	< 0.001	67.8 (13.3)	71.3 (15.1)	< 0.001
Body mass index at pregnancy start (kg/m^2^)	52,678	23.7 (4.0)	24.8 (4.6)	< 0.001	23.7 (3.9)	24.7 (4.5)	< 0.001	24.1 (4.4)	25.4 (5.1)	< 0.001
Age at menarche (years)	52,025	13.1 (1.4)	12.9 (1.3)	< 0.001	13.1 (1.4)	12.9 (1.4)	< 0.001	13.0 (1.4)	12.8 (1.4)	< 0.001
Years from menarche to pregnancy (years)	26 901^b^	16.0 (4.5)	15.5 (4.4)	0.008	16.2 (4.4)	15.7 (4.5)	0.001	14.6 (4.4)	14.2 (4.5)	< 0.03
	*n*	*n (%)*	*n (%)*		*n (%)*	*n (%)*		*n (%)*	*n (%)*	
Parity	52,678									
Primiparous	27,758	8329 (63.8)	696 (47.8)	< 0.001	8203 (48.5)	1263 (34.2)	< 0.001	7741 (55.5)	1526 (42.1)	< 0.001
Maternal education (years)	52,678									
≤ 12	16,045	3790 (29.1)	547 (37.6)	< 0.001	4375 (25.9)	1266 (34.3)	< 0.001	4597 (32.9)	1470 (40.5)	< 0.001
Smoking during pregnancy	52,320									
Daily	2685	823 (6.4)	157 (10.9)	< 0.001	633 (3.8)	191 (5.2)	< 0.001	636 (4.6)	245 (6.8)	< 0.001
Incidences of irritability before menses	47,565									
Yes	29,497	6479 (52.4)	775 (58.2)	< 0.001	9984 (63.4)	2350 (70.5)	< 0.001	7817 (61.8)	2092 (66.9)	< 0.001
Previous experience of NVP	21 639^c^									
Yes	7792	347 (8.5)	85 (14.7)	< 0.001	1682 (22.5)	629 (32.4)	< 0.001	3731 (65.4)	1318 (70.1)	< 0.001

^a^These include PGP and sPGP during pregnancy, as well as PGP and sPGP 4-6 months post-partum

^b^Primiparous women

^c^Multiparous women

When the total study sample was compared with women excluded from the sample, similar estimates were seen in regards to age, weight, BMI, and age at menarche, but among the excluded women a lower proportion were primiparous (48.4% vs 52.7%, found in supporting information Additional file 1), had ≤12 years of education (29.2% vs 30.5%), and a higher proportion were daily smokers (5.7 vs 5.1%).

### Time of NP, NVP, and PGP symptom initiation

**Fig. 1:**
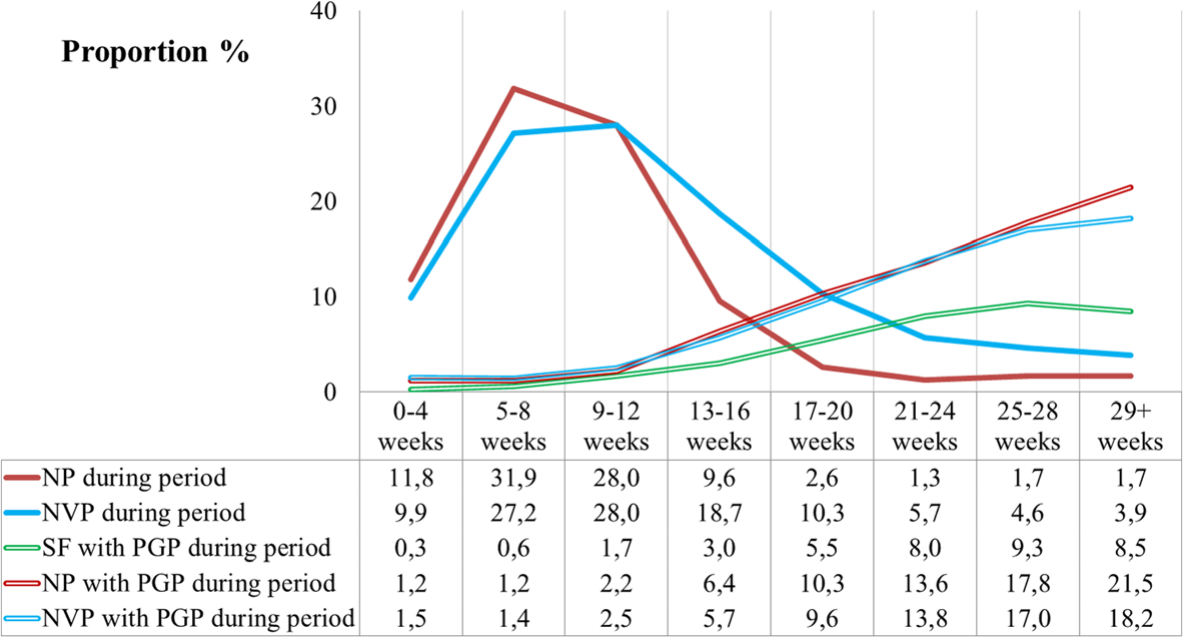
Proportions of women with nausea alone (NP) or nausea and vomiting in pregnancy (NVP) in each period, and proportions with pelvic girdle pain (PGP) among symptom-free (SF), NP, and NVP women in each period, *n* = 52,678

When we studied the timing of onset of total NP and NVP during the specific periods in pregnancy, we observed a sharp increase in early first trimester, with NP peaking in weeks 5-8 (31.9%) and NVP in weeks 9-12 (28.0%, Fig. 1). Thereafter, both conditions decreased steadily. By contrast, among the SF, NP and NVP women, the proportions of PGP increased gradually from 0.3, 1.2 and 1.5%, in weeks 0-4, respectively, to 8.5, 21.5 and 18.2%, respectively, in week 29 or over.

### Duration of nausea and vomiting in relation to PGP

**Fig. 2:**
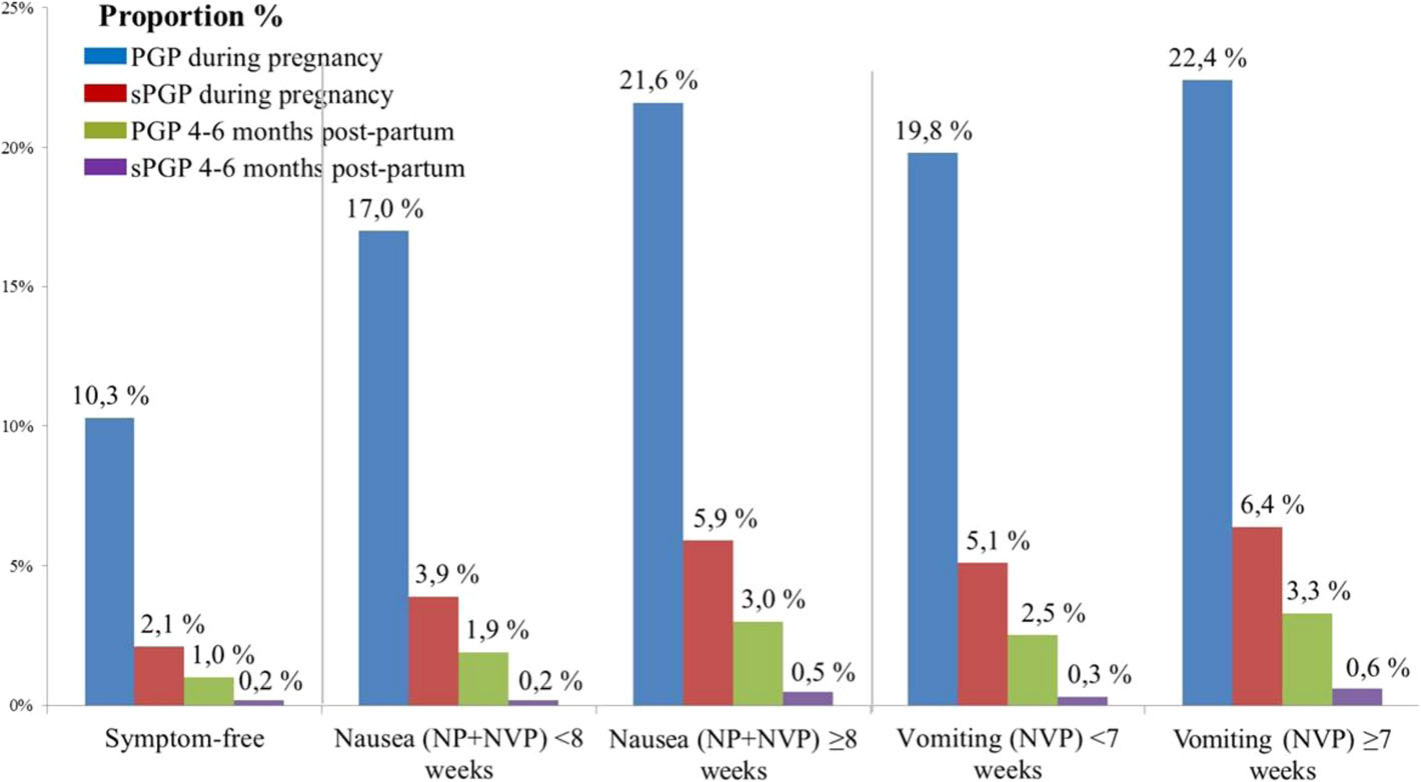
Proportions of pelvic girdle pain (PGP) and severe PGP (sPGP) during pregnancy and 4-6 months post-partum stratified by median duration of nausea and vomiting for nausea alone (NP) and nausea and vomiting during pregnancy (NVP) women, *n* = 46,766

When comparing the occurrences of PGP and sPGP during pregnancy and 4-6 months post-partum with shorter or longer periods of nausea or vomiting, we observed consistently higher proportions of PGP and sPGP for women with longer durations of either nausea or vomiting (Fig. 2).

### Association between NVP and PGP

Analysis of women with PGP and sPGP 4-6 months post-partum showed the NVP group having the highest proportion as compared to the NP and SF groups (Table 2).

**Table 2:** Crude and adjusted odds ratios (cOR, aOR) of pelvic girdle pain (PGP) and severe PGP (sPGP) 4-6 months post-partum (pp) in relation to group (symptom-free (SF), nausea only (NP), and nausea and vomiting (NVP)), *n* = 46 662^a^ (13,126 SF women, 18,384 NP women, and 15,152 NVP women)

			Crude		Adjusted^b^	
Outcome	Group	n (%)	cOR (95% CI)	*P* value	aOR (95% CI)	*P* value
PGP at 4-6 months pp	SF	95 (0.7)	1.00	< 0.001	1.00	< 0.001
	NP	308 (1.7)	2.34 (1.86-2.95)		2.14 (1.70-2.71)	
	NVP	334 (2.2)	3.09 (2.46-3.89)		2.83 (2.25-3.57)	
sPGP at 4-6 months pp	SF	12 (0.1)	1.00	< 0.001	1.00	< 0.001
	NP	34 (0.2)	2.03 (1.05-3.91)		1.87 (0.96-3.64)	
	NVP	54 (0.4)	3.91 (2.09-7.31)		3.35 (1.78-6.32)	

^a^Women with values in all primary and adjusted for variables

^b^Adjusted for maternal age, body mass index, smoking during pregnancy, parity, education, age at menarche, and irritability before menses

Compared to SF women, NP and NVP women had significantly higher odds of PGP 4-6 months post-partum (aOR = 2.14, 95% CI 1.70-2.71, and aOR = 2.83, 95% CI 2.25–3.57, respectively). NVP women also had significantly higher odds of sPGP 4-6 months post-partum (aOR = 3.35, 95% CI 1.78-6.32) while non-significantly increased odds were found among NP women (aOR = 1.87, 95% CI 0.96-3.64).

## Discussion

We found that women with both NVP and PGP were the heaviest, and had the highest proportion with an education ≤12 years. PGP with either NP or NVP appeared later in pregnancy compared to symptoms of NP or NVP only. Women with a longer duration of nausea or vomiting reported PGP and sPGP more frequently compared to women with a shorter duration. In multivariate analyses, women with NP and NVP had higher odds than SF women of experiencing PGP and sPGP at 4-6 months post-partum.

Women excluded from the study had similar age at delivery, weight, BMI, and ages at menarche. Furthermore, there was a lower proportion of primiparous women (4.3%) amongst the excluded, which is consistent with non-compliance reported elsewhere [34]. The higher proportions of daily smokers in the excluded group (0.6%) may be a result of the high number of missing values (23%). Furthermore, it has been presented elsewhere that although prevalence estimates such as smoking are under-represented in the MoBa cohort when compared with the general Norwegian population, the exposure-outcome associations are valid [35]. The deviation (1.3%) regarding maternal education may also be a reflection of the high number of missing cases in the excluded group (22%).

Gestational week 9-16 is the crossover period in which the proportions of NP and NVP decreased and the proportions of PGP increased amongst the women. As our data indicate that NP and NVP symptoms are more prevalent before PGP symptoms appear, and the proportion of women with PGP is higher for those having the longer duration of NP or NVP, it seems plausible to speculate that the presence of NP or NVP may exacerbate the occurrence of PGP.

NVP and PGP appear to have many risk factors in common. In this study we report that a prior pregnancy, high BMI, lower number of years from menarche to pregnancy, and incidences of irritability before menses, were all more present for the women experiencing NP and NVP with PGP.

Many of these risk factors may be attributable to hormonal mechanisms. A higher BMI has previously been shown to have a strong inverse association with the sex hormone-binding globulin and the total estrogen levels in the large prospective Nurses’ Health Study [36]. Additionally, numerous studies have suggested a hormonal aetiology in relation to NVP symptoms, yet no consensus has been achieved. Progesterone and estrogen/estradiol in high or low concentrations have been proposed previously [37–39], although others could not reproduce these findings [40, 41]. This situation is also the case for NVP’s most hypothesised causative hormone, hCG [38, 41–43].

It has previously been hypothesised that abnormal serum levels of relaxin during pregnancy increase the risk of PGP, although findings are inconsistent [44, 45]. Relaxin affects collagen synthesis during pregnancy, and together with progesterone, has been suggested to influence joint laxity in pregnant women [46]. In addition to relaxin, significantly higher levels of progesterone in early pregnancy have been suggested to play a role in the development of PGP [26]. The relationship between NVP and PGP identified in this study may be based on a synergistic effect of pregnancy hormones such as progesterone, estrogen and relaxin, although it remains speculative without access to early pregnancy biomarkers. Relaxin has apparently never been investigated clinically in relation to NVP symptoms, which is remarkable when one considers that both hCG and relaxin tend to peak in concentration at approximately the same time during early pregnancy [47, 48].

Our new results regarding PGP and sPGP 4-6 months post-partum strengthen our previous findings suggesting NP and NVP women have increased odds for pregnancy-related PGP [24]. One study of women experiencing PGP post-partum reported that pain was exacerbated by the onset of menstruation and/or ovulation [49], which further implies a hormonal contribution in PGP.

### Strengths and limitations

The strengths of this study are the large sample from a population-based cohort and the linkage to the MBRN, providing detailed access to maternal health during pregnancy. The MoBa cohort specifically addressed NVP-related issues, providing the opportunity to differentiate between NP and NVP women [5, 24, 33]. Likewise, the detailed questions regarding PGP formed a basis for the robust definition of PGP used [21]. As this is a large cohort, numerous significant associations tend to appear, yet the merit of these in the clinical setting are not always relevant. Furthermore, the responses in the MoBa questionnaires do not allow for an easy assessment of the severity of NVP symptoms. Retrospective evaluation of NVP symptoms has previously been reported as a possible source of bias [50]. We attempted to address this by excluding cases with inconsistencies in NVP symptoms reported between questionnaires (*n* = 15,791) to have as much confidence as possible in the remaining study sample. When we performed sensitivity analyses with the excluded cases included in the sample, the results were mostly unaffected. Women excluded from the present study had similar ages at delivery, BMI and energy intake, while there tended to be more women with a lower education (29.2% vs 30.5%) as well as a higher parity (48.4% vs 52.7%), findings consistent with non-compliance present in other studies [34, 51, 52]. The slightly higher number of daily smokers in the excluded group (5.7% vs 5.1%) is most likely a product of the high number of missing values for this variable. Other weaknesses include the reliance on self-reports of PGP, nausea, and vomiting. The diagnosis of PGP in our study would have benefited from a clinical assessment, as has been suggested elsewhere [13], in order to support the women’s self-reports. However, this is not feasible in a large cohort study. Categorising the difference between retching and actual vomiting (defining NP and NVP) may also have led to misclassifications and affected the results. Another weakness is the lack of information on biomarkers (e.g. hormones).

## Conclusions

We found that women with NP or NVP had higher odds of experiencing PGP or sPGP 4-6 months post-partum, compared to SF women. To our knowledge this is the first study examining elements of nausea and vomiting in relation to PGP post-partum. NP and NVP appeared earlier in pregnancy than PGP, and women with a longer duration of nausea and/or vomiting had a higher proportion of PGP compared to those women with a shorter duration of symptoms, suggesting a synergistic relationship between NP/NVP and PGP.

Our findings showing NP or NVP women with PGP representing a high proportion with irritability before menses and a lower number of years from menarche to first birth compared to those groups without PGP tend to support the hypothesis of a hormonal involvement in the underlying mechanisms of these disorders.

## Additional file

Additional file 1: Selected maternal characteristics for women included and excluded from the study. (DOCX 19 kb)

## Abbreviations

aOR: Adjusted odds ratio
BMI: Body mass index
CI: Confidence interval
cOR: Crude odds ratio
hCG: human chorionic gonadotropin
HG: Hyperemesis gravidarum
MBRN: Medical birth registry of Norway
MoBa: The Norwegian Mother and Child Cohort Study
NP: Nausea alone
NVP: Nausea and vomiting in pregnancy
PGP: Pelvic girdle pain
Q1-4: Questionnaire number
SD: Standard deviations
SF: Symptom-free
sPGP: severe pelvic girdle pain
