# Supplementary material for: Nausea and vomiting in pregnancy – association with pelvic girdle pain during pregnancy and 4-6 months post-partum
Source: BMC Pregnancy Childbirth. 2018 May 8;18:137. doi: 10.1186/s12884-018-1764-7 (PMC5941485; doi:10.1186/s12884-018-1764-7)
Supplement: Supplementary file 1 — Selected maternal characteristics for women included and excluded from the study. (DOCX 19 kb) [file 12884_2018_1764_MOESM1_ESM.docx]

**Additional file 1.**

**Selected maternal characteristics for women included and excluded from the study, *n* = 95 507^1^**

|  | **n** | **Included**  **(*n* = 52 678)** | **Excluded**  **(*n* = 42 829)** | ***n*** |
| --- | --- | --- | --- | --- |
| Maternal age at delivery (y) | 52 678 | **Mean (SD)**  30.1 (4.6) | **Mean (SD)**  29.7 (4.9) | 42 829 |
| Maternal weight (kg) | 52 678 | 67.9 (12.8) | 67.7 (13.0) | 31 829 |
| BMI^2^ at pregnancy start (kg/m²) | 52 678 | 24.0 (4.3) | 24.0 (4.3) | 31 829 |
| Age at menarche (years) | 52 025 | 13.0 (1.4) | 13.1 (3.0) | 33 740 |
|  | | **n (%)** | **n (%)** |  |
| Parity  Primiparous | 52 678 | 27 758 (52.7) | 20 711 (48.4) | 42 829 |
| Maternal education (y)  ≤12 y  Missing | 52 678 | 16 045 (30.5)  1105 (2.1) | 12 490 (29.2)  9474 (22.1) | 42 829 |
| Smoking during pregnancy  Daily  Missing | 52 678 | 2685 (5.1)  358 (0.7) | 2452 (5.7)  9967 (23.3) | 42 829 |

^1^114 275 children – multiple births (n=3971 children) = 110 304 single children/women

110 304 women – 52 678 women included = 57 626 excluded women.

57 626 excluded women – ≥1 MoBa participation (n=14 797 duplicate women) = 42 829 excluded women

^2^Body mass index
